# Report of the Foreign Relations Committee of the National Association of Dental Faculties

**Published:** 1900-10

**Authors:** 


### REPORT OF THE FOREIGN RELATIONS COMMITTEE OF THE NATIONAL ASSOCIATION OF DENTAL FACULTIES.[1]

During the past year the work of the Foreign Relations Committee has been materially extended. Advisory boards in most foreign countries have been provided for, and appointments made to fill them as fast as sufficiently definite information to enable the committee to do this properly could be obtained. Pamphlets containing an exposition of the work and the aims of the National Association of Dental Faculties have been printed and circulated in foreign countries, and a number of circulars of information for members of our foreign advisory boards have been printed and mailed to them. In addition, as directed by the Association at its last meeting, a pamphlet containing digests of the reports made at that meeting has been printed and mailed to each member of the Association, and to other interested members of the profession in America and abroad.

All this has involved considerable expense for printing and post-

[1] Reported and adopted at Old Point Comfort, Va., July 14, 1900.

age, but we believe that it has been a wise expenditure of money, as by its means the dental profession of the world has been made aware of the existence of an association of the regular and recognized dental schools of America which is devoted to the advancement of the cause of dental education and to the elevation of the status of dentistry among all nations.

It is unfortunately the fact that, because of the lack of uniformity in the educational systems of the different States, and the absence of any general supervisory authority on the part of the national government, under some unwise local legislation it has been possible for irresponsible, unqualified, and unscrupulous men to secure charters for institutions empowered to grant degrees, and under such authority to issue, for a consideration, irregular and fraudulent diplomas. This traffic has principally been with men in foreign countries, who, primarily the guilty ones, have sought to obtain academic honors without the labor necessary honestly to acquire them. As these institutions have been conducted under pretentious names, it was formerly impossible for foreigners who had no intimate acquaintance with American educational affairs to distinguish between the regular and the irregular schools. The organization of this Association has established a criterion by which they may be judged, only those owning allegiance to the National Association of Dental Faculties being recognized.

It is unfortunate that the professional situation in America has not in past years been better comprehended in Europe. All our schools have been held responsible for the vile work of the fraudulent ones,—nominally located in this country, but chiefly supported by unprofessional men from abroad. There has even been a grave misapprehension of the objects of this Association, and the work of the Foreign Relations Committee has in some instances been totally misconstrued. All of us are aware that while some of the very best and ablest American representatives have located in foreign countries, and to whose professional career we can point with pride, it is unfortunately the case that some Americans of a different professional reputation have gone abroad and have indulged in practices as offensive to our foreign *confrères* as they are to reputable American practitioners. There are many more unworthy foreigners who have legitimately or illegitimately become possessed of an American degree, and who, without warrant of right, claim the title of "American dentist."

The belief is prevalent in certain foreign professional circles that it is the aim of the National Association and its Foreign Relations Committee to obtain for all such persons professional recognition, and to demand the acceptance of their American degree by the governments of foreign countries. It is but proper that we should in the most authoritative manner deny any aspirations of the kind. This Association has not in the remotest manner contemplated any interference with or protest against the laws or regulations governing the practice of dentistry in any foreign country. It has not primarily been the object of either the National Association or its Foreign Relations Committee to attempt to secure for the American dental degree any legal recognition as a qualification for foreign practice. It is not usual in the American States which have legal professional regulations to receive the diplomas of any foreign professional school as a qualification for practice, and we cannot consistently ask that which we refuse to others.

It seems but proper that we should publicly avow the reasons that have prompted the better colleges to form this association of schools, and to appoint a committee charged with the duty of harmonizing our relations with the dental profession in other lands. We seek for the distinctive American dental diploma nothing more than the consideration which its merits demand. If its reputation has been debased by the circulation of counterfeit diplomas, it is something for which we are in no way responsible. In the formative educational period, when dental schools existed nowhere save in America, and when even dentistry itself was undefined, empirical, tentative, with no distinctive line of practice and no clearly prescribed curriculum of study, the newly adopted degree may have been conferred in some instances on insufficient acquirements. The experiment of establishing a special dental educational course of study, and thus laying the foundation for the broad profession which exists in all civilized countries to-day, was first tried in America, and here tested for the whole world. There were no precedents for our guidance, and no earlier successes or failures to stand as landmarks. We were the absolute pioneers, and it would be little wonder if we made some errors.

Since that day other countries have drawn professional lines, and marked out, each for itself, a distinctive course of procedure. Each of these somewhat varies from the others, and perhaps all from that originally established in America. If dentistry is to be accepted as a profession at all, or as a distinct branch of a great mother profession, it must be broader than is any State; it cannot be confined by any bourne, nor limited by mountains, rivers, or oceans. There should be no American, English, German, or French dental profession, except as each is a part of one undivided whole. Realizing all this, the National Association of Dental Faculties was organized for, and has been constantly laboring to attain, these definite purposes:

*First.* To establish a broad and generally accepted curriculum of dental study, and by the combination of all the better dental schools of America to bring each up to a uniform standard of excellence.

*Second.* To establish a clear line of demarkation between the regular and the irregular schools, and to force out of existence the latter.

*Third.* Gradually to raise the standard of preliminary education until none but such as have the general erudition that should distinguish a professional man can be accepted in American dental colleges.

These were the principal objects in view, and in the attainment of them success has been secured exceeding the most sanguine expectations of the founders of the movement.

In the development of its plans the Association met with many obstacles, and found itself laboring under great embarrassments. One of the chief of these was the lack of information concerning professional affairs in foreign countries. The Association decided, so far as was in its power, to co-operate with the worthy dentists of other countries in the laying down of certain broad principles which must be the foundation upon which any true professional practice could rest. Any international co-operation must be based upon a complete knowledge by each of the methods and aims of the others. There can be no concurrent effort without mutual comprehension and intelligence.

Another perplexity was found in the fact that in establishing the preliminary qualifications for matriculation in American colleges there was no rule by which to judge of the value of certificates presented by foreign students. After completing the course of some foreign school, a student, who perhaps spoke only a strange language, sometimes desired to conclude his studies by taking as much of the American course as would enable him to finish it, and he demanded of some American college advanced standing of one or more years. His certificates were in a foreign tongue, and in some instances were found either forged or not that which they were represented to be.

In this emergency, at the earnest request of certain American dentists practising in foreign countries, who had been scandalized by the acceptance in America of students with improper certificates, a committee, to be called the "Committee on Foreign Relations," was appointed, and was charged with certain definite duties:

*First.* It was to be in all things subordinate and subservient to the National Association of Dental Faculties, to which body it must make a full report each year.

*Second.* It was empowered to appoint advisory boards of not more than three members in each foreign country having any professional relations with America, whose reports concerning foreign qualifications might form a basis for action in this country.

*Third.* It was to have jurisdiction in all foreign educational questions affecting American dental colleges.

*Fourth.* It was to obtain definite information concerning dental regulations and laws in foreign countries; to learn what were the curriculum and requirements of all foreign dental schools, with the view of determining what value should, under American laws and regulations, be given their certificates of study, either as a qualification for dental practice in America or for admission to advanced standing in American dental colleges.

*Fifth.* It was charged with the duty of ferreting out institutions engaged in the granting of irregular degrees or degrees irregularly, and instituting measures for their suppression.

In compliance with the first enumerated duty your committee makes this report of what it has done during the past year, and appends the recommendations for future action which its experience leads it to believe advisable. It has earnestly striven to carry out what its members believed to be the wishes of this Association, and it has had no policy of its own to inaugurate or attempt to enforce. It has in all things been governed by what it believed to be the spirit of its instructions.

Concerning the second business with which it was charged, your committee begs to report that it has divided the various countries of both the eastern and western hemispheres into convenient groups, and has appointed boards for each, so far as the information obtainable has warranted. In making such appointments it has deemed the following qualifications essential:

*First.* The appointee should be a regular and reputable dentist, possessing the legal qualifications of the country which he represents.

*Second.* He must be a graduate of some reputable American dental school, or possess an acquaintance with the curricula of American schools, and be familiar with American dental professional methods. The list of such appointments is appended for the approval of this Association.

In the discharge of the third duty imposed upon us your committee has met with great embarrassments. At the very outset colleges, members of this Association, appealed to us to know what consideration should be given to certificates showing that proposed students had taken the full course in schools located in Japan and Mexico, which purported to teach the whole dental curriculum. Your committee could not learn that any schools giving a course in dentistry that could be accepted as an equivalent for any part of that demanded by this Association existed in either country. They therefore ruled that students from either could only be accepted as members of the freshman class of American dental colleges, and only then if they complied with the rules of the Association so far as preliminary education and a knowledge of the English language are concerned. This ruling was cheerfully accepted by the schools that had raised the question, and we present it as an encouraging proof of the loyalty and anxious desire for a high standard that exists among the recognized dental colleges of America.

But the discussion of this raised the question of the consideration that should be given to the certificates of study from any foreign dental school. Our rules provide that no credit shall be given to certificates from any American dental school whose curriculum and regulations have not received the formal approval of this Association. Could we, in the name of the National Association of Dental Faculties, approve the giving of advanced standing to students from the schools of other countries that had not the same stamp of regularity? That is, could we extend to foreign and unknown dental teaching institutions privileges that were positively forbidden to American schools? And yet the responsibility of deciding this question had been thrust upon us by this Association, and we could not evade the obligation. It took but a short time to arrive at the inevitable conclusion that we could not approve the giving of advanced standing to graduates or undergraduates of any foreign dental school whatever until such school had received the formal indorsement of this body.

Fortunately, few of these questions arose in time to affect any student for the term of 1899-1900. We informed the colleges presenting the cases that the matter would be referred to this annual meeting, and the committee is prepared to offer certain recommendations for the recognition of foreign schools, based upon such knowledge as we have been able to obtain. The whole matter is referred to this body for final adjustment.

In the discharge of the fourth duty that devolved upon us, your committee is in possession of a very voluminous mass of correspondence and reports, which it has earnestly labored to reduce to some system. The advisory boards appointed have, in a considerable number of instances, forwarded as full information concerning dental schools and the regulations governing dental practice in the countries represented by them as could be obtained, and it is upon such reports that the recommendations of your committee are wholly based. How much of them shall be given to the profession of America by publication must be decided by the Association. It would be quite impossible to print the great mass of correspondence unless a large volume should be devoted to that purpose.

Under the fifth head, your committee begs leave to report that a great deal has been accomplished. The same legal counsel employed last year has been retained, and the same general course has been pursued. It is probable that more fraudulent diplomas have been sold in foreign countries during the past year than ever before. This is due to the fact that those who have been carrying on the traffic realize that, because of activity in their prosecution, the time for accountability is near at hand, and they are striving to make the most of the present opportunity.

It is urged by foreigners that this business should be summarily stopped. Such people little know the difficulties in the way. In the first place, the traffic is mostly with foreigners. As their illegitimate diplomas are wholly worthless in this country, no State Board of Examiners recognizing them in any way, those who are engaged in the business carefully cover their tracks, and no responsible man can be located. Attempts to entrap them by means of decoy letters have failed, some such having crossed the ocean a number of times without delivery, being forwarded from one of their foreign agents through whom the nefarious business is carried on to another, until finally returned to the writer by the post-office authorities. Fictitious names are signed to the pretended diplomas, so that it has been found almost impossible to fix the guilt upon any person. Our friends in foreign countries have contented themselves with bitter reproaches against American colleges generally, without forwarding any testimony that would assist in the discovery of the guilty ones. The fraudulent institutions could not by foreigners be distinguished from the regular colleges, for they were in possession of charters regularly granted under a vicious law of the State of Illinois, whose entire repeal it had been found impossible to secure, because the interests of legitimate enterprises were inextricably bound up with the illegitimate ones.

Your committee early discovered that working alone it could accomplish little. The Board of Health of the State of Illinois was taking the matter up, and they possessed advantages for the prosecution of the lawbreakers which were not within our reach. We have therefore contented ourselves with co-operating with that board in every way possible, and our counsel has been instructed to offer them any assistance within our power. As a consequence we have great pleasure in reporting that, acting under the United States law, which forbids the use of the mails for fraudulent purposes, the worst of these offenders have finally been apprehended and committed to jail in defaut of the heavy bail that was demanded. What is of more importance, if possible, the United States mails are closed against the transmission of their correspondence, and letters to or from them are promptly sequestrated.

The greatest offender was last year named in this report as "The Independent Medical College of Chicago." We secured the annulment of the charter of this affair, but in a very short time we found that the same men were yet engaged in the business under the name of "The Cosmopolitan Medical College." They had offered for sale no less than thirty-six different diplomas in all the branches of science and art, and since the forfeiture of the charter under which they first worked it is believed they have sold more than a thousand fraudulent diplomas, at prices varying from ten to five hundred dollars each. Proof sufficient to secure the cancellation of the first charter was only obtained through the inordinate cupidity of the man who was chiefly responsible. He paid a debt of some thirty dollars due to a stable-man, or hostler, by issuing a diploma to him and making him a professional man. The recipient, when he found himself under arrest for attempting practice under it, betrayed the swindler, and we were thus able to fix his guilt.

The late proceedings against this man and his associates have developed the fact that they were in possession of no less than *twenty-four* different charters, all regularly issued under that mischievous Illinois law, which was enacted for beneficent purposes. We have now learned the methods of these men, and it is believed that it will soon be possible to put an entire stop to their villanous traffic, through the imprisonment under the United States postal laws of those engaged in it. Too much credit cannot be given the Board of Health of the State of Illinois for the active part it has taken in the suppression of these miserable pretenders that have so long been bringing discredit upon our legitimate and excellent educational institutions.

In view of the fact that the other work of the Foreign Relations Committee is more than sufficient to engage all its surplus energies, and in further consideration that the work of the suppression of the fraudulent schools is now well in hand and the path for action fully defined, your committee recommends that this work be, for the future, placed in the hands of the Committee on Law, which shall receive the same instructions as those heretofore given the Committee on Foreign Relations.

The progress that this Association is making in its efforts to raise the status of professional teaching in our own country, to obtain a better appreciation of American professional affairs in foreign countries, and to maintain steady advancement towards a dental solidarity among all nations is very encouraging to every lover of humanity. It is true that even at home there may in uninformed circles yet be found some remnants of an unworthy professional jealousy, a failure to comprehend the real educational situation, and a tendency to attribute to our teachers motives unworthy any honest man. But the steady, persistent work of this Association in elevating the accepted standard just as fast as prudence permits, has wrought a great change in professional sentiment and immeasurably benefited the schools, and through them the profession at large. It only remains for us to continue this good work a few years longer to produce results that will be permanent in their character, and so firmly established as henceforth to be self-sustaining.

REPORT CONCERNING FOREIGN EQUIVALENTS.

Your committee has very carefully considered a great mass of correspondence and many voluminous reports, and begs hereby to submit the conclusions which it has reached. It must not be forgotten that the system of dental instruction in Europe varies very widely from that of our special American dental schools. Instruction separate from that given in the medical schools or universities is very rare, and the practical training which forms a part of our curriculum is usually given by private preceptors.

Your committee does not feel at liberty to recommend the acceptance of an oral and theoretical course as the equivalent for one including practical work. We cannot believe that the certificates of private and irresponsible practitioners can by us be accepted as any part of a college course, and hence we have given them little consideration. It is quite probable that in some instances we have recommended that one year's advanced standing be given the holders of some certificates when further knowledge might show that they should be admitted to our senior classes, but we have thought it wisdom to err, if any mistakes are made, upon the safer side, as future action can readily correct any such errors.

*Australia.*

A very complete report from the various colonies of Australia and New Zealand has been made by the advisory board appointed for those countries. It would appear that in most of the colonies there is no dental legislation, but Victoria has lately secured a law analogous to that of England, and in Melbourne a dental school has been organized whose curriculum, from the partial syllabus furnished, seems to be a comparatively broad one. The dean of the "Australia College of Dentistry" is an American graduate, and he appears to have the confidence of the dentists of Australia.

Your committee is unable positively to determine whether the school in all respects comes up to our minimum requirements, but this it has directed its chairman definitely to ascertain, after which your committee will be prepared to recommend to this body some proper action. There has also been established in Melbourne, Province of Victoria, the "Dental College and Oral Hospital of

Victoria," but your committee is not at the present time in possession of sufficiently definite information to enable it to offer any recommendation concerning it.

In the provinces of Western Australia and Tasmania no dental legislation has been secured.

There is a dental law in New Zealand, and the member of the advisory board from that province has furnished your committee with an abstract of it. There are no dental schools in the province.

*Switzerland.*

Full reports from this country have been furnished by Dr. Bryan. It is a republic analogous to our own country in some respects, the federal union being composed of separate cantons. There are some excellent universities which offer certain facilities for dental study, but their practical instruction, we believe, cannot be accepted as an equivalent for that offered by American dental colleges. Your committee recommends that holders of the Swiss national diploma be given one year's advanced standing in the schools of this Association, but that no consideration be at present extended to holders of the cantonal qualifications.

*Spain.*

Complete reports have been furnished by members of the advisory board. The Spanish requirements in medicine are very high, but your committee cannot learn that there are any dental schools, or dental departments of universities, whose course of instruction can be accepted as the full equivalent for the instruction given in American dental colleges.

*France.*

Your committee is aware that separate dental schools exist in France, and its chairman has been in daily expectation of receiving their curriculum of study, but up to this time has been disappointed. Without this exact knowledge the members do not feel themselves justified in recommending any action, for we cannot proceed in so grave a matter upon mere assertions or impressions. As members of your committee will visit France in the immediate future, and will carefully investigate the course of study, we ask that we be given authority to incorporate our recommendations in this report after such investigation shall have been completed.

*Germany and Austria.*

The dental schools of these countries are departments of the universities, and only university students attend them. The instruction consists of lectures and clinical work given by from one to three dental professors, who lecture upon the different dental subjects. Instruction in chemistry and allied studies is afforded in the School of Philosophy or Science; in anatomy, physiology, etc., in the School of Medicine. No special instruction is given dental students except by the very few dental teachers. The clinical instruction is largely devoted to extraction and oral surgery. The practical work is usually quite limited. There is no obligatory course, but students enter for such lectures as they may choose, paying the fees of each professor separately. There are no obligatory hours for study or lectures.

The mechanical instruction consists of lectures on the principles of mechanics, the practical work being usually done in private laboratories. The examinations have very little resemblance to ours, each teacher asking three questions out of a list of forty approved by government. They are not usually as exhaustive or comprehensive or scrutinizing as ours. The licensing or approving power rests with the "Kultus Ministerium," or department of religion and education. The great majority of dentists in practice are Zahntechnichers,—mechanical dentists,—upon whose work no restrictions are placed, as they are not recognized by the government.

Your committee recommends that students speaking the English language, who have taken the full dental course in German or Austrian universities, be eligible for reception in the junior classes of American dental colleges, provided it be shown that they have had at least two semesters of competent college instruction in practical laboratory and operative work. It further recommends that students speaking the English language who have had at least four semesters of such instruction in operative and prosthetic practical courses, and who shall have finished the dental course in the University of Berlin, or in any German or Austrian dental school whose course of instruction offers a full equivalent, be eligible for admission to the senior classes of accepted American dental colleges.

### *Italy.*

In Italy the practice of dentistry was long without special restrictions. Then an attendance upon lectures in a medical school was required, and a dental diploma was issued. In 1892 a law was passed which required dentists to obtain a medical diploma. This was not enforced until 1898, when a movement against foreign practitioners was inaugurated. They appealed to the courts and carried the matter to the supreme court, which decided that those in practice previous to 1888 had rights which could not be abrogated. At present the law of 1892 is in force, and this requires a medical diploma for the practice of dentistry and phlebotomy.

There are, we believe, no schools in Italy which have courses that can be accepted as equivalent to those of our American dental schools. The instruction given in the medical schools your committee believes to be too exclusively general in its character to form an acceptable course in dentistry for American students.

### *Mexico.*

There is a medical school in the City of Mexico which purports to give dental instruction. Your committee cannot learn that it is of such a character as will enable it to be accepted as the equivalent for a course in an American college.

### *Japan.*

There is one dental school in Japan,—that of Dr. Takayama, in Tokio. It confers no degree, but gives a certificate which entitles the holder to government examination, the same as if he had studied with some practising dentist. As the instruction is personal and the school is quite irresponsible, your committee believes that no consideration can be given to it.

### *Holland and Belgium.*

In these countries the title of dentist is obtained by passing a practical examination in the theory and practice of dentistry. There are no separate dental schools, and we are not sufficiently informed of the comprehensiveness of the syllabi of the universities to offer any recommendations concerning them.

### *Great Britain.*

There can be no questioning the fact that England has some excellent dental schools. The only embarrassing circumstance in the determination of their status relative to ours lies in the great difference between the educational systems of the two countries. Undoubtedly they place greater stress upon preliminary educational requirements than do we, but your committee is of the opinion that our practical instruction is superior. Originally, we believe, there was little instruction given in prosthetic work during the term of attendance upon hospital lectures. Students were supposed to come to the college for didactic instruction, the practical part having been previously communicated by a preceptor. It should be comprehended that English dentists frequently employ a mechanic, who is not required to possess any special educational qualifications, the registered dentist mainly confining his attention to the operations of the surgery or operating-room.

In this country we believe the practical work of the laboratory should form a part of the college course, and we do not graduate a student until he shall have satisfactorily completed the whole curriculum within the college walls. We are under the impression that the English system is undergoing a change in this respect, and that practical laboratory work will soon form a part of the obligatory college course. We recommend that all students who shall have finished the complete course in any recognized English, Irish, or Scotch dental school or hospital shall be eligible for reception as senior students in American dental colleges upon proof of their having taken as a part of such course two years of instruction in a properly equipped dental laboratory and dental infirmary connected or affiliated with such dental school or hospital, and which requires the successful completion of the work deemed essential by recognized American schools, as formulated in the minimum requirements for foreign dental schools accompanying this report. We further recommend that for the present no consideration be given to partial courses in any of the dental schools of Great Britain.

*Sweden.*

Very complete reports have been furnished by the chairman of the advisory board, Dr. Förberg.

The country has one dental school, which is the dental department of the "Carolina Medico-Chirurgical Institute of Stockholm." Instruction is given by five professors of the medical department, and there are three dental professors, occupying respectively the chairs of dental surgery, operative dentistry, and dental prosthetics and orthodontia. From the assurances given by Dr. Förberg, your committee believes that its graduates should be permitted to enter the second-year class of recognized American dental colleges, provided they shall have complied with our requirements concerning mechanical laboratory work.

Your committee has not sufficient knowledge concerning this school to warrant further recommendations at present.

*Canada.*

In the Dominion of Canada there is but one school which demands consideration, and that is a member of this body. Yet the educational systems of the two countries, especially in professional matters, are so different as to engender continual embarrassments. Canada being a foreign country, your committee has felt itself bound in duty to place it in the list of those countries whose relations with us must be taken into consideration. The dental educational system of Ontario approaches more nearly that of England than that of America. It has an analogous system of indentures which the dental student must sign, and private preceptorship forms a portion of its obligatory instruction.

This is directly at variance with our system, which accepts no tutorship by irresponsible parties. The dental law of Ontario forbids the entrance upon practice of any one who has not taken his final course of instruction in the Royal College of Dental Surgeons of Ontario. We believe that this principle is the correct one, and that the same rule should be made applicable in the United States, and that here, as there, no foreign qualification should be sufficient for registration in the various States of America. But the membership of this foreign school in our Association presents an embarrassment which for the present seems insuperable, and your committee therefore has no recommendation to make, but leaves the matter for future consideration in the hope that some code of international agreement may be devised which will give to the graduates of America's recognized colleges who desire to practise in Canada the same privileges extended to the alumni of the excellent Ontario dental college.

Concerning other foreign countries, your committee is not in possession of sufficiently definite information to warrant any action. We have no knowledge of the existence of any courses of instruction which can be accepted as an equivalent for courses in institutions having membership in this body, and therefore advanced standing in our schools cannot in justice to our own students be granted, save in the instances above enumerated. The committee will gladly make use of any further information which may be furnished them, and will, in furtherance of the duty with which they are charged by this Association, embody such knowledge in future reports.

REPORT CONCERNING THE MINIMUM REQUIREMENTS TO BE DEMANDED BY THE NATIONAL ASSOCIATION OF DENTAL FACULTIES FOR THE RECOGNITION OF FOREIGN DENTAL SCHOOLS WHOSE STUDENTS DESIRE ADVANCED STANDING IN THE COLLEGES BELONGING TO THE ASSOCIATION.

1. The college must require of matriculants a preliminary education which is the full equivalent of that demanded by the schools of this Association.

2. The college must demand of students full attendance upon at least three full annual courses (not semesters) of lectures of not less than seven calendar months each, in separate years, covering all the studies proper to a full dental curriculum.

3. The college must possess a bacteriological laboratory, with sufficient of equipment for instruction in a competent course in bacteriology, which must form a part of its curriculum of study.

4. The same must be required in chemistry, histology, and pathology.

5. There must be a technic laboratory in which shall be taught the proper manipulations for the insertion of all kinds of fillings for teeth, the preparation and filling of the roots of teeth, the tempering and shaping of instruments, the drawing of wire and tubing for cases in orthodontia, and the cutting of bolts and nuts.

6. There must be prosthetic laboratories sufficiently equipped for teaching all kinds of prosthetic work, and the construction of all the approved prosthetic appliances.

7. There must be a sufficiently equipped laboratory for instruction in making crowns and bridges, and the construction of appliances used in orthodontia.

8. There must be a properly equipped infirmary or surgery for the reception of patients, upon whom each and every student shall be required individually to perform all and enough of the operations necessary in dental practice thoroughly to qualify him for the successful pursuance of his profession.

9. Complete records of the work done by each student, of his attainments at sufficient and full examination in each subject of the curriculum of study, of his attendance and deportment during the course, must be permanently kept.

10. No credit must be allowed for any work not done under the immediate supervision of instructors connected with or especially approved by the college, and who are in direct affiliation with the faculty.

The following is a list of the countries for which advisory boards have been designated, and the appointments and nominations so far as made:

| Country. | Name. | College. | Post-Office Address. |
|---|---|---|---|
| Great Britain. | Wm. Mitchell, D.D.S. | Univ. of Michigan. | 39 Upper Brooks Street, London, England. |
| Great Britain. | W. E. Royce, D.D.S. | Phil. Dental College. | 2 Lonsdale Gardens, Tunbridge Wells, England. |
| Great Britain. | B. J. Bonnell. | ...................... | 94 Cornwall Gardens, S. Kensington, London. |
| Holland and Belgium. | J. E. Grevers, D.D.S. | ...................... | 13 Oude Turfmarkt, Amsterdam, Holland. |
| Holland and Belgium. | Ed. Rosenthal, D.D.S. | Harvard Univ. | 19 Boul. du Regent, Brussels, Belgium. |
| Holland and Belgium. | C. Van der Hoeven, D.D.S. | ...................... | Der Haag. |
| Denmark, Swe., & Nor'y. | Elof Förberg, D.D.S. | Phil. Dental College. | Sturegatan 24, Stockholm, Sweden. |
| Denmark, Swe., & Nor'y. | S. S. Andersen, D.D.S. | Univ. Pennsylvania. | Christiana, Norway. |
| Denmark, Swe., & Nor'y. | L. P. Vorslund-Kjaer, D.D.S. | Phil. Dental College. | Copenhagen, Denmark. |
| Russia. | H. V. Wollison, D.D.S. | N. Y. Coll. Dent. | 10 Quai de l'Amaranti, St. Petersburg, Russia. |
| Russia. | Theo. Weber, D.D.S. | N. Y. Coll. Dent. | Helsingfors, Finland. |
| Russia. | Geo. Th. Berger, D.D.S. | Phil. Dental Col. '77. | St. Petersburg, Russia. |
| Germany. | W. D. Miller, D.D.S. | Univ. Pennsylvania. | Victoriastrasse 30, Berlin, Germany. |
| Germany. | C. F. W. Bödecker, D.D.S. | N. Y. Coll. Dent. | 55 Unter den Linden, Berlin, Germany. |
| Germany. | Friedrich Hesse, D.D.S. | N. Y. Coll. Dent. | Goethe Str. 6, Leipsig, Germany. |
| Austria and Hungary. | Dr. Szigmondi. | ...................... | ...................... |
| Austria and Hungary. | Dr. Wacisser. | ...................... | ...................... |
| Austria and Hungary. | Dr. Arkövy. | ...................... | ...................... |
| Italy and Greece. | Albert T. Webb, D.D.S. | Univ. Pennsylvania. | 87 Via Nazionale, Rome, Italy. |
| Italy and Greece. | Tullio Avanzi. | ...................... | ...................... |
| Italy and Greece. | A. V. Elliott, D.D.S. | Univ. of Mich. '87. | 10 Via Tornabuoni, Florence, Italy. |
| France. | J. H. Spaulding D.D.S. | Univ. Minnesota. | 39 Boul. Malesherbes, Paris, France. |
| France. | I. B. Davenport, M.D. | Coll. P. and S., New York. | 30 Ave. de l'Opera, Paris, France. |
| France. | G. A. Roussell, D.D.S. | N. Y. Coll. Dent. | 74 B'd Haussmann, Paris, France. |

| COUNTRY. | NAME. | COLLEGE. | POST-OFFICE ADDRESS. |
|---|---|---|---|
| Spain and Portugal. | R. H. Portuondo, D.D.S. | Univ. Pennsylvania. | Paseo de Recoletos 3, Madrid, Spain. |
| Spain and Portugal. | Florestan Aguilar, D.D.S. | Phil. Dental Coll. | Serrano 5, Madrid, Spain. |
| Spain and Portugal. | T. J. Thomas, D.D.S. | ........................ | Bilbao, Spain. |
| Switzerland and Turkey. | L. C. Bryan, D.D.S. | Boston Dental Coll. | 1 Steinenberg, Basel, Switzerland. |
| Switzerland and Turkey. | Theo. Frick, D.D.S. | Univ. Pennsylvania. | 14 Tonhallenstrasse Zurich, Switzerland. |
| Switzerland and Turkey. | Paul J. Guye, D.D.S. | Penn. Dental Coll. | 12 Rue de Candolle, Geneva, Switzerland. |
| Japan, China, and India. | Louis Ottofy, D.D.S. | Western Dental Coll. | 87 Main Street, Yokohama, Japan. |
| Japan, China, and India. | J. Ward Hall, D.D.S. | ........................ | Shanghai, China. |
| Japan, China, and India. | ........................ | ........................ | ........................ |
| Australia & New Zealand. | Alfred Burne, D.D.S. | Phil. Dental Coll. | 1 Lyons Terrace, Liverpool Street, Sydney. |
| Australia & New Zealand. | A. P. Merrill, D.D.S. | Phil. Dental Coll. | 52 Collins Street, Melbourne. |
| Australia & New Zealand. | Herbert Cox, D.D.S. | Univ. of Michigan. | 216 Queen Street, Aukland, New Zealand. |
| Cuba & W. India Islands. | ........................ | ........................ | ........................ |
| Cuba & W. India Islands. | Rice R. Buchanan, D.D.S. | ........................ | 47 San Francisco Street, San Juan, Porto Rico. |
| Cuba & W. India Islands. | ........................ | ........................ | ........................ |
| Mexico & Cent. America. | ........................ | ........................ | ........................ |
| Mexico & Cent. America. | ........................ | ........................ | ........................ |
| Mexico & Cent. America. | ........................ | ........................ | ........................ |
| Venez., Colom., & Ecua'r. | ........................ | ........................ | ........................ |
| Venez., Colom., & Ecua'r. | ........................ | ........................ | ........................ |
| Venez., Colom., & Ecua'r. | ........................ | ........................ | ........................ |
| Peru, Bolivia, and Chili. | S. R. Salazar, D.D.S. | Chicago Coll. Dental Surgery. | Lima, Peru. |
| Peru, Bolivia, and Chili. | ........................ | ........................ | ........................ |
| Peru, Bolivia, and Chili. | ........................ | ........................ | ........................ |
| Brazil and Guiana. | ........................ | ........................ | ........................ |
| Brazil and Guiana. | ........................ | ........................ | ........................ |
| Brazil and Guiana. | ........................ | ........................ | ........................ |
| Argentine, Para., & Uru. | ........................ | ........................ | ........................ |
| Argentine, Para., & Uru. | ........................ | ........................ | ........................ |
| Argentine, Para., & Uru. | ........................ | ........................ | ........................ |

W. C. BARRETT, *Chairman*,
208 Franklin Street, Buffalo, N. Y.

S. H. GUILFORD,
1728 Chestnut Street, Philadelphia, Pa.

J. D. PATTERSON,
Ninth and Walnut Streets, Kansas City, Mo.

T. W. BROPHY,
126 State Street, Chicago, Ill.

H. W. MORGAN,
211 N. High Street, Nashville, Tenn.

*Foreign Relations Committee.*

